# Effects of Dietary Supplementation with Mushroom or Vitamin D_2_-Enriched Mushroom Powders on Gastrointestinal Health Parameters in the Weaned Pig

**DOI:** 10.3390/ani11123603

**Published:** 2021-12-20

**Authors:** Alison Dowley, Torres Sweeney, Eadaoin Conway, Stafford Vigors, Supriya Yadav, Jude Wilson, William Gabrielli, John V. O’Doherty

**Affiliations:** 1 School of Agriculture and Food Science, University College Dublin, Belfield, D04 V1W8 Dublin, Ireland; alison.dowley@ucdconnect.ie (A.D.); eadaoin.conway@ucdconnect.ie (E.C.); staffordvigors1@ucd.ie (S.V.); 2 School of Veterinary Medicine, University College Dublin, Belfield, D04 V1W8 Dublin, Ireland; torres.sweeney@ucd.ie; 3 MBio, Monaghan Mushroom Group, Tyholland, Co., H18 FW95 Monaghan, Ireland; supriya.yadav@mbio.ie (S.Y.); jude.wilson@mbio.ie (J.W.); william.gabrielli@gmail.com (W.G.)

**Keywords:** pigs, mushroom, vitamin D, inflammation, microbiota, β-glucan, zinc oxide, *Agaricus* *bisporus*, gut health

## Abstract

**Simple Summary:**

The prospective ban on zinc oxide in pig feed in Europe is a major challenge facing the swine industry to maintain piglet health and performance during the weaning period. Weaning is a particularly difficult period for the young pig that is associated with abrupt dietary, environmental and social changes that cause significant levels of stress and disrupt gut development in the pig. Mushrooms are a rich natural source of bio-actives and have long been regarded as a health-promoting food due to their immunomodulatory and antioxidant effects and their ability to modulate the gut microbiota. Mushrooms contain high levels of ergosterol, which allows them to naturally produce vitamin D when they are exposed to light. The present study aimed to compare the effects of mushroom and vitamin D_2_-enriched mushroom powders to zinc oxide on the molecular, physiological and microbial changes that influence performance during the post-weaning period. Our study showed that vitamin D_2_-enriched mushrooms were equally as effective as zinc oxide in improving gastrointestinal health parameters. However, both mushroom powders reduced feed intake in pigs and negatively affected animal performance. For this reason, mushroom powders have limited use as a commercial feed additive in replacing zinc oxide in pig diets.

**Abstract:**

The objective of this study was to compare the molecular, physiological and microbial effects of mushroom powder (MP), vitamin D_2_ enriched mushroom powder (MPD_2_) and zinc oxide (ZnO) in pigs post-weaning. Pigs (four pigs/pen; 12 pens/treatment) were assigned to: (1) basal diet (control), (2) basal diet + ZnO, (3) basal diet + MP (2 g/kg feed) and (4) basal diet + MPD_2_ (2 g/kg feed). Zinc oxide supplementation improved the feed intake (*p* < 0.001); increased the caecal abundance of *Lactobacillus* (*p* < 0.05); increased the villus height (*p* < 0.05) in the duodenum, jejunum and ileum; increased the expression of chemokine interleukin 8 (*CXCL8*; *p* < 0.05); and decreased the expression of pro-inflammatory cytokine gene interleukin 6 (*IL6*; *p* < 0.05), tumour necrosis factor (*TNF*; *p* < 0.05), nutrient transporters peptide transporter 1 (*SLC15A1*; *p* < 0.05) and fatty acid binding protein 2 (*FABP2*; (*p* < 0.05) in the duodenum. Whereas dietary supplementation with MPD_2_ improved the gastrointestinal morphology (*p* < 0.05); increased the total volatile fatty acid concentrations (*p* < 0.05); increased the expression of anti-inflammatory cytokine gene interleukin 10 (*IL10*; *p* < 0.05) and nutrient transporters *SLC15A1* (*p* < 0.05), *FABP2* (*p* < 0.05) and vitamin D receptor (*VDR*; *p* < 0.05); and reduced the expression of pro-inflammatory cytokine gene IL6 (*p* < 0.05), it adversely affected average daily feed intake (ADFI; *p* < 0.001) and average daily gain (ADG; *p* < 0.05). Mushroom powder supplementation had a positive impact on gastrointestinal morphology (*p* < 0.05) and upregulated the expression of nutrient transporters *SLC15A1* (*p* < 0.05) and *FABP2* (*p* < 0.05) and tight junction claudin 1 (*CLDN1*) (*p* < 0.05) compared to the controls but had no effect on the expression of inflammatory markers (*p* > 0.05). Furthermore, MP reduced ADFI (*p* < 0.01); however, this did not negatively impact the ADG (*p* > 0.05). In conclusion, MP and MPD_2_ have limited use as commercial feed additives in replacing ZnO in pig diets as feed intake was reduced post-weaning.

## 1. Introduction

Pharmacological doses (2000–3100 mg/kg feed) of zinc oxide (ZnO) are often included in the post-weaning pig diet to alleviate the negative impacts of weaning on pig performance [1] and gastrointestinal health [2,3,4]. Common protocols employed on commercial farms include feeding 3100 mg/kg ZnO for 2 weeks post-weaning, followed by 1550 mg/kg for 3 additional weeks. However, high levels of ZnO supplementation have been associated with lasting changes in the composition of the gastrointestinal microbiota, such as a decreased abundance of the genus *Lactobacillus* [5] and increased selection for antimicrobial resistant bacteria [6].

Thus, the European Union (EU) have begun the phasing out of pharmacological doses of ZnO alongside further restrictions on the use of infeed medication in weaner pig diets by 2022 (Commission Implementing Decision of 26 June 2017, C(2017) 4529 Final) (Regulation (EU) June 2019). Therefore, there is increasing pressure in identifying natural bioactive compounds that may effectively reduce gastrointestinal inflammation and dysbiosis similar to that of ZnO whilst maintaining profitability and animal performance. Two bioactives of interest are β-glucans and Vitamin D, due to their well-recognized immunomodulatory properties.

Beta-glucans with β-(1 → 3, 1 → 6) linkages are the major structural components of fungal and yeast cell walls [7]. The key biological activities of fungal and yeast β-glucans include anti-inflammatory, antioxidant and immunomodulatory effects [8,9,10], and as they are a non-digestible polysaccharide, they can positively influence the gut microbiota through increased *Lactobacillus* and *Bifidobacteria* populations [11,12].

Vitamin D has conventionally been associated with bone mineralisation and calcium homeostasis; however, more recent research has demonstrated vitamin D to play an important role in the modulation of inflammation by stimulating the expression of antimicrobial peptides, reducing the production of inflammatory cytokines, such as tumour necrosis factor (TNF) and interleukin 6 (IL-6) and increasing the production of anti-inflammatory cytokine interleukin 10 (IL-10) [13].

Vitamin D plays an essential role in maintaining a healthy epithelial barrier and gut microbiota, through the reduction of pathogenic bacterial species, including Escherichia coli [14]. Vitamin D can be synthesised in the body upon exposure to ultraviolet B (UVB) light [15]; however, in a typical commercial setting, pigs are raised indoors and, therefore, must obtain vitamin D from their diet.

Mushrooms offer a unique opportunity to naturally incorporate β-glucans and vitamin D into the diet of the pig and are a natural, cost-effective and sustainable source of vitamin D and β-glucans. Mushrooms, such as white button mushrooms (*Agaricus bisporus*) are also a rich natural source of biologically active metabolites, such as phenolics, lectins, terpenoids and ergosterols [16,17]. Mushrooms can naturally produce vitamin D as they contain high levels of the vitamin D precursor ergosterol, which has the potential to form a bioavailable dietary source of vitamin D when exposed to UVB light post-harvest [18].

Dietary supplementation with vitamin D_2_-enriched mushroom powder improved ADG and FCR in 150-day old finisher pigs [19]; however, the same effect was not observed in younger pigs [20]. Therefore, the objective of this study was to compare the molecular, physiological, and microbial effects of mushroom powder (MP), vitamin D_2_-enriched mushroom powder (MPD_2_) and zinc oxide (ZnO) in these younger pigs.

## 2. Materials and Methods

In this study, all procedures were approved under the University College Dublin Animal Research Ethics Committee, Ireland (AREC-18-27-O’Doherty) and were conducted in accordance with Irish legislation (SI no. 543/2012) and the EU directive 2010/63/EU for animal experimentation. All efforts were taken to minimize pain and discomfort to the animals while conducting these experiments.

### 2.1. Experimental Design, Animal Management and Diets

At weaning (28 days), 192 pigs (progeny of Meatline boars × (Large White × Landrace sows)) with an average weight of 7.8 kg (SD 1.07) were selected from a commercial pig farm. The pigs were blocked by weaning weight, sex and litter of origin and, within each block, assigned to one of four treatment groups. The dietary treatments were as follows: (1) Basal diet (control); (2) basal diet + ZnO; (3) basal diet + unenriched mushroom powder (MP) containing a β-glucan content of 200 mg/kg feed; and (4) basal diet + vitamin D_2_ enriched mushroom powder (MPD_2_) containing a β-glucan content of 200 mg/kg feed and an additional vitamin D_2_ content of 100 µg/kg feed.

The ZnO was included at 3100 mg ZnO/kg feed and contained 80% zinc, resulting in an inclusion level of 2500 mg Zn/kg feed. After 15 days the inclusion level of ZnO was halved to 1550 mg ZnO/kg feed and then completely removed from the diet on day 35 for a period of 10 days pre-slaughter. The concentration of β-glucans (200 mg/kg) was selected based on previous studies [21,22]. The dried mushroom powder (*Agaricus bisporus*) was sourced from Monaghan Mushrooms, Ireland, and 2 g/kg was included to achieve the β-glucan inclusion level of 200 mg/kg.

The mushroom powder contained 305 g/kg of crude protein, 34 g/kg of total fat, 100 mg/g of β-glucan and 4 µg/g of vitamin D prior to UVB exposure. The vitamin D mushroom powder contained a vitamin D concentration of 50 ug/g mushroom powder. The mushroom vitamin D content was naturally enhanced following UVB exposure, as previously described [23]. The ZnO was sourced from Cargill (Naas, Kildare, Ireland). The diets were formulated to have equivalent net energy (10.6 MJ/kg), crude protein (208 g/kg) and standardised ileal digestible lysine (13.0 g/kg). All diets contained a vitamin D_3_ content of 50 µg/kg feed. All amino acid requirements were calculated relative to lysine [24]. The compositions of the diets are presented in Table 1.

### 2.2. Housing and Animal Management

The pigs were penned in groups of four according to weight and sex and housed on fully slatted floors (1.68 m × 1.2 m). There were 12 replicate pens used per treatment with two male and two female pigs in each replicate pen. For the first 7 days, the temperature within the weaner house was controlled at 30 °C and then reduced by 2 °C per week until the temperature reached 22 °C.

The humidity was maintained at 65%. The experimental diets were given in meal form, and the pigs had ad libitum access to these diets immediately after weaning up to the final weighing. Clean drinking water was available ad libitum from a drinking nipple. Pigs received no medication throughout the experiment. Body weight (BW) was measured using a portable electronic scale (Prattley, Temuka, New Zealand) on days 1 and 45 and the average daily gain (ADG) and average daily feed intake (ADFI) were calculated.

Faecal scores were assessed twice daily for each individual pen to indicate the presence and severity of diarrhoea. The following scoring system was used to assign faecal scores: 1 = hard, 2 = slightly soft, 3 = soft, partially formed, 4 = loose, semi-liquid and 5 = watery, mucous like [26]. For diarrhoea incidence, a faecal score of greater than 3 was categorised as diarrhoea.

### 2.3. Feed Analysis

All the feed samples were milled through a 1 mm screen (Christy and Norris Hammer Mill, Chelmsford, UK) and kept for chemical analysis. The gross energy (GE) content was determined using an adiabatic bomb calorimeter (Parr Instruments, Moline, IL, USA) as previously described [27]. The feed was dried overnight at 104 °C to determine the dry matter (DM) content of the feed. Feed samples were analysed for crude ash, nitrogen, crude fibre, acid detergent fibre (ADF) and ether extract according to the Association of Official Agricultural Chemists standard procedures [28].

Neutral detergent fibre (NDF) was determined according to the method of Van Soest et al. [29]. The total glucans of the MP were determined using the kit K-YBGL, purchased from Megazyme (Bray, Ireland), following the manufacturer’s recommendations and as previously described [30]. The vitamin D content of the vitamin D_2_-enriched MP was established using high performance liquid chromatography, as previously described [31]. All samples were measured in duplicate.

### 2.4. Sample Collection

On day 45 of the experiment, one pig/pen that was randomly selected at weaning time (48 pigs; 24 male and 24 female; 12 pigs/treatment) was euthanized at a rate of 0.71 mL/kg body weight with pentobarbitone sodium (Euthatal Solution, 200 mg/mL; Meri-al Animal Health, Essex, UK), at a rate of 0.71 mL/kg body weight (BW) injected into the cranial vena cava. Sections from the duodenum, jejunum and ileum were processed for gut morphological analysis as previously described [32].

Digesta from the colon and caecum was collected and stored in sterile containers (Sarstedt, Wexford, Ireland). This was then snap frozen on dry ice and stored at −80 °C for subsequent 16 s rRNA sequencing and volatile fatty acid (VFA) analysis. In addition, tissue samples were taken from the duodenum, jejunum and ileum to measure the expression of cytokines, nutrient transporters, mucins, tight junctions and appetite regulators using quantitative real-time PCR (QPCR).

Tissue sections (1 cm) from the duodenum, jejunum and ileum were cut out, dissected along the mesentery, emptied and rinsed using sterile phosphate buffered saline (Oxoid, Hampshire, UK). The tissue sections were stripped of the overlying smooth muscle before storage in RNAlater^®^ (5 mL) solution (Applied Biosystems, Foster City, CA, USA) overnight at 4 °C. The RNAlater^®^ was removed before storing the samples at −80 °C.

### 2.5. Gut Morphological Analysis

Standard paraffin embedding techniques were used to prepare the small intestinal tissue for gut morphological analysis as previously described [32]. A light microscope with an image analyser (Image-Pro Plus; Media Cybernetics, Oxon, UK) was used to measure the villus height and crypt depth. Fifteen measurements of villi and crypt were taken for each section.

### 2.6. Gene Expression in the Small Intestine

#### 2.6.1. RNA Extraction and cDNA Synthesis

The total RNA was extracted from duodenal and ileal tissues using TRI Reagent (Sigma-Aldrich, St. Louis, MO, USA) in accordance with the manufacturer’s guidelines as previously described [33]. The total RNA (2 μg) was reverse transcribed using a High-Capacity cDNA Reverse Transcription Kit (Applied Biosystems, Foster City, CA, USA) and oligo (dT) primers in a final reaction volume of 40 μL, in accordance with the manufacturer’s guidelines. The cDNA was then made up to a volume of 360 μL with nuclease-free water.

#### 2.6.2. Quantitative Real-Time Polymerase Chain Reaction (QPCR)

The qPCR reaction mixture (20 μL) consisted of GoTaq qPCR Master Mix (10 μL) (Promega, Madison, WI, USA), forward and reverse primers (5 μM), (1.2 μL), nuclease-free water (3.8 μL) and cDNA (5 μL). All the QPCR reactions were conducted in duplicate on the 7500 ABI Prism Sequence detection System (Applied Biosystems, Foster City, CA, USA). The cycling conditions consisted of a denaturation step of 95 °C for 10 min, which was followed by 40 cycles of 95 °C for 15 s and then 60 °C for 1 min. All the primers were designed using the Primer Express Software (Applied Biosystems, Foster City, CA, USA) and made by MWG Biotech UK Ltd. (Milton Keynes, UK) and are all described in Table 2. Dissociation curves were created to verify the specificity of the subsequent PCR products.

The QPCR assay efficiencies were determined by plotting the cycling threshold (CT) values resulting from four-fold serial dilutions of cDNA against their arbitrary quantities, and only assays demonstrating 90–110% efficiency and single products were accepted in this analysis. Normalised relative quantities were obtained using the software, qbase PLUS (Biogazelle, Ghent, Belgium) from stable reference genes; *B2M*, *GAPDH* and *YWHAZ* (duodenum) and *B2M* and *PPIA* (ileum). These genes were selected as reference genes based on their M value (<1.5) generated by the GeNorm algorithm within GeNorm.

The genes analysed in the current study were as follows: protein transporter *SLC15A1* (previously known as *PEPT1*); fatty acid transporter *FABP2*; vitamin D receptor *VDR*; glucose transporters *SLC2A1* (previously known as *GLUT1*), *SLC2A2* (previously known as *GLUT2*) and *SLC2A5* (previously known as *GLUT5*); sodium-glucose transporter *SLC5A1* (previously known as *SGLT1*); appetite regulator *CCK*; cytokines *TNF*, *IL6*, *IL10*, *IFNG*, *TGFB1* and *IL17*; chemokine *CXCL8* (previously known as *IL8*); mucins *MUC2* and *MUC1*; tight junctions *CLDN3*, *CLDN1* and *ZO1*; Toll like receptor *TLR4*; and reference genes *B2M*, *GAPDH*, *YWHAZ* and *PPIA*.

### 2.7. Volatile Fatty Acid Analysis

Gas liquid chromatography was used to determine the VFA concentrations in the colonic digesta, as described in detail by Clarke et al. [34]. We diluted 1 g of digesta with water (2.5× sample weight) and centrifuged (1400× *g* for 10 min) using a Sorvall GLC-2B centrifuge (DuPont, Wilmington, DE, USA). We mixed 1 mL of supernatant and 1 mL of internal standard (0.05% 3-methyl-n-valeric acid in 0.15 M oxalic acid dihydrate) with 3 mL of distilled water and then centrifuged for 10 min at (500× *g*).

The supernatant was then filtered through a syringe filter (0.45 polytetrafluoroethylene (TFE)) into a chromatographic sample vial. Approximately 1 µL of this mixture was injected into a Varian 3800 GC (Markham, ON, Canada) with an ECTM 1000 Grace column (15 m × 0.53 mm I.D) with a film thickness of 1.20 µm. The temperature program was set to the range 75–95 °C, which increased by 3 °C/min and 95–200 °C, which increased by 20 °C/min, and this was held for 0.50 min. The detector temperature was 280 °C, and the injector temperature was 240 °C. The total analysis time was 12.42 min.

### 2.8. Microbiological Analyses

#### 2.8.1. Microbial DNA Extraction

A QIAamp PowerFecal Pro DNA Kit (Qiagen, West Sussex, UK) was used to extract microbial genomic DNA in accordance with the manufacturer’s instructions. A Nanodrop ND-1000 Spectrophotometer (Thermo Scientific, Wilmington, DE, USA) was used to measure the quantity and quality of the DNA.

#### 2.8.2. Illumina Sequencing

Bacterial DNA was extracted from the caecal digesta samples and high-throughput sequencing of the V3–V4 hypervariable region of the bacterial 16S rRNA gene was performed on an Illumina MiSeq platform according to their standard protocols (Eurofins Genomics, Ebersberg, Germany).

#### 2.8.3. Bioinformatic and Statistical Analysis

The bioinformatic assessment of the sequences were conducted by Eurofins Genomics (Ebersberg, Germany) using the package (Version 1.9.1) Quantitative Insights into Microbial Ecology [35]. All the raw reads passing the standard Illumina chastity filter were demultiplexed in accordance with their index sequences (read quality score > 30). The primer sequences were clipped from the beginning of the raw forward/reverse reads. If primer sequences did not match perfectly, read pairs were eliminated to retain only high-quality reads.

Paired-end reads were then merged, to obtain a single, longer read that covers the complete target region using the software FLASH 2.2.00 [36]. The pairs were merged with the lowest overlap size of 10 bp to decrease false-positive merges. The forward read was only kept for the subsequent assessment steps when merging was not viable. Merged reads were quality filtered in accordance with the expected and known length variations of the V3–V4 region (ca. 445 bp).

The ends of retained forward reads were clipped to a complete read length of 285 bp to eliminate low quality bases. Merged and retained reads comprising of ambiguous bases were removed. The filtered reads were then used for profiling of the microbiome. Chimeric reads were detected and deleted based on the de-novo algorithm of UCHIME [37] as implemented in the VSEARCH package [38]. The remaining set of high-quality reads were then processed using minimum entropy decomposition (MED) to partition reads to operational taxonomic units (OTU) [39,40].

DC-MEGABLAST alignments of cluster representative sequences to the NCBI nucleotide sequence database were conducted for the taxonomic assignment of every OTU. A sequence identity of 70% across a minimum of 80% of the representative sequence was the minimal prerequisite for considering reference sequences. The abundances of bacterial taxonomic units were normalized using lineage-specific copy numbers of the appropriate marker genes to enhance estimates [41].

The data matrix was made up of the normalized OTU table in combination with the phenotype metadata and phylogenetic tree. The data matrix was then loaded into the phyloseq package in R (http://www.r-project.org; Version 3.5.0, accessed on 2 September 2020). The dynamics of richness and diversity in the microbiota were computed with the observed, Chao1, ACE, Shannon, Simpson, inverse Simpson and Fisher indices. The Simpson and Shannon indices of diversity accounted for richness and evenness parameters. The beta diversity measurements are a calculation of separation of the phylogenetic structure of the OTU in one sample compared with all the rest of the samples.

This was estimated by normalizing the data so that taxonomic feature counts were comparable across all the samples. Numerous distance metrics were considered to calculate the distance matrix of the various multidimensional reduction processes. These included weighted and unweighted UniFrac distance/non-phylogenetic distance metrics (i.e., Bray–Curtis, Jensen–Shannon divergence and Euclidian) using phyloseq within R [42,43].

Differential abundance analysis was conducted on tables extracted from the phyloseq object at the phylum, family, genus and species level. The PROC Glimmix procedure within Statistical Analysis Software (SAS) 9.4 was used to analyse the data. The model examined the effect of ‘treatment’, with the individual pig being the experimental unit. For the statistical analysis of the relative bacterial abundances, 12 pigs per treatment were used. The results are presented using Benjamini–Hochberg (BH) adjusted *p*-values.

All data on growth performance, gastrointestinal morphology, gene expression and VFA were checked for normality using the univariate procedure of Statistical Analysis Software (SAS) 9.4. The general linearized model (GLM) procedure within SAS was used to analyse the data on growth performance, gastrointestinal morphology, gene expression and VFA. The model examined the effect of treatment and using weight at weaning as a covariate.

Faecal scores were averaged each week for the first 14 days and analysed using the PROC MIXED procedure of SAS. The incidence of diarrhoea during the first 14 days post weaning was analysed using the PROC Genmod procedure of SAS. For the growth performance data, the pen was the experimental unit, whilst for the gastrointestinal morphology, gene expression and VFA data, the pig was the experimental unit. The probability level that denoted significance was *p* < 0.05. The data are presented as least-square means and standard error of the mean.

## 3. Results

### 3.1. Growth Performance, Faecal Scores and Mortality Rates

The MPD_2_ supplemented pigs had lower ADG and ADFI (*p* < 0.05) compared with the ZnO and control groups (Table 3). The MP supplemented pigs had a lower ADG (*p* < 0.05) compared with the ZnO group and a lower ADFI (*p* < 0.05) compared with the ZnO and control groups. The ZnO supplemented pigs had reduced faecal scores (*p* < 0.05) and diarrhoea incidence (*p* < 0.001) compared with all other groups. There was no difference (*p* > 0.05) in the faecal scores or diarrhoea incidence between the MP, MPD_2_ and control groups. There was an overall 2% mortality rate; however, these deaths were unrelated to the experimental diets.

### 3.2. Small Intestinal Morphology

In the duodenum, pigs supplemented with MP, MPD_2_ or ZnO had increased VH (*p* < 0.05) compared with the control group (Table 4). In the jejunum, pigs supplemented with MPD_2_ and ZnO had increased VH (*p* < 0.05) compared with the control group. Pigs offered MP had decreased CD (*p* < 0.05) in the jejunum compared with the ZnO and control groups. Pigs supplemented with MP and MPD_2_ had increased VH:CD (*p* < 0.05) in the jejunum compared with the control group.

In the ileum, pigs supplemented with MPD_2_ and ZnO had increased VH (*p* < 0.05) compared with the control group. Pigs offered MP had decreased CD (*p* < 0.05) in the ileum compared with the control and ZnO groups. Pigs supplemented with MP and MPD_2_ had increased VH:CD (*p* < 0.05) in the ileum compared with the control group.

### 3.3. Effects of Mushroom Powder Supplementation on the Caecal Microbiota

#### 3.3.1. Bacterial Richness and Diversity

Dietary supplementation had no effect on the Observed, Chao1, ACE, Shannon, Simpson, InvSimpson and Fisher index measures of alpha diversity (*p* > 0.05) in the caecal digesta (Table 5).

#### 3.3.2. Differential Bacterial Abundance Analysis

All data on bacterial abundances at phylum, family and genus level are presented in Table 6, Table 7 and Table 8. There were five bacterial phyla identified with Firmicutes being the dominant phyla (~67.3%) followed by Bacteriodetes (~30.6%), Proteobacteria (~0.73%), Actinobacteria (~0.59%) and Tenericutes (~0.09%). There was no effect of dietary supplementation on the measures of relative abundance at the phylum level (*p* > 0.05; Table 6).

At family level, dietary supplementation with MP and MPD_2_ decreased (9.92 and 10.32 vs. 14.16, SEM 1.000, *p* < 0.05) the relative abundance of Lactobacillaceae within the Firmicutes phylum compared with the ZnO group (*p* < 0.05; Table 7). Dietary supplementation with ZnO decreased (27.84 vs. 33.10, SEM 0.074, *p* < 0.05) the relative abundance of Prevotellaceae within the Bacteroidetes phylum (*p* < 0.05) compared with the control group.

At the genus level, dietary supplementation with MP and MPD_2_ decreased (9.92 and 10.32 vs. 14.16, SEM 1.000, *p* < 0.05) the relative abundance of Lactobacillus within the family Lactobacillaceae (*p* < 0.05) compared with the ZnO group (Table 8). Dietary supplementation with ZnO, MP and MPD_2_ decreased (23.38, 24.90 and 23.94 vs. 29.06 SEM 1.451, *p* < 0.05) the relative abundance of Prevotella within the family Prevotellaceae (*p* < 0.05) compared with the control group. Of the taxa attributed to the families Lactobacillaceae, Propionibacteriaceae and Burkholderiaceae, Lactobacillus, Propionibacterium and Ralstonia, respectively, were the only genus identified.

### 3.4. Volatile Fatty Acids

Pigs supplemented with MPD_2_ had increased total colonic VFA’s compared with all other groups (*p* < 0.05; Table 9). Supplementation with MPD_2_ reduced the molar proportions of isobutyrate compared with the control group and reduced the molar proportions of isobutyrate, butyrate and isovaline compared with the ZnO supplemented pigs.

### 3.5. Gene Expression in the Small Intestine

The complete gene expression data is presented in Table 10. Genes that are differentially expressed in the small intestine are highlighted in this section:

#### 3.5.1. Nutrient Transporter Gene Expression

In the duodenum, pigs supplemented with MP and ZnO had increased expression of peptide transporter *SLC15A1*, (*p* < 0.05) and fatty acid transporter *FABP2*, (*p* < 0.05) compared with the control group. Pigs supplemented with MPD_2_ had increased expression of *SLC15A1*, (*p* < 0.05) and *FABP2*, (*p* < 0.05) in the duodenum compared with the control group and an increased expression of vitamin D receptor *VDR*, (*p* < 0.05) in the duodenum compared with the control and ZnO group. In the ileum, pigs supplemented with ZnO had reduced expression of glucose transporter 2 (*SLC2A2*, *p* < 0.05) compared with the control group.

#### 3.5.2. Expression of Genes Involved in Inflammation and the Epithelial Barrier

In the duodenum, pigs supplemented with MPD_2_ had reduced expression of chemokine *CXCL8*, (*p* < 0.05) compared with the ZnO group and reduced expression of cytokine gene *IL6*, (*p* < 0.05) in the duodenum compared with the control group. Pigs supplemented with ZnO had reduced expression of cytokine genes *TNF*, (*p* < 0.05) and IL6, (*p* < 0.05) and increased expression of *CXCL8*, (*p* < 0.05) in the duodenum compared with the control group.

In the ileum, pigs supplemented with MPD_2_ had increased expression of cytokine gene *IL10*, (*p* < 0.05) compared with the control and ZnO group. Pigs supplemented with MP had reduced expression of *CXCL8*, (*p* < 0.05) in the ileum compared with the MPD_2_ group. Pigs supplemented with MP had increased expression of tight junction gene *CLDN1*, (*p* < 0.05) in the ileum compared with all other groups.

## 4. Discussion

The positive response observed with ZnO supplementation, including improved feed intake; increased villus height in the duodenum, jejunum and ileum; and positive effects on immune markers, nutrient transporters, faecal and diarrhoea incidence; and a higher abundance of *Lactobacillus* in the caecal microbiota, supports the credible role of ZnO in maintaining gastrointestinal homeostasis.

The MP enhanced gastrointestinal morphology and integrity and increased the expression of nutrient transporters in the duodenum, while MPD_2_ supplementation improved the gastrointestinal morphology, increased the expression of nutrient transporters and exhibited anti-inflammatory effects. Despite the beneficial effects of MP and MPD_2_ on the markers of gut health, supplemented pigs had reduced ADFI and ADG.

Maximizing the feed intake in newly weaned pigs is critical, as low feed intake immediately post-weaning negatively impacts nutrient uptake and utilization, subsequently leading to impaired gut structure and function and reduced performance [44]. The MP and MPD_2_ supplementation reduced the feed intake compared with the control and ZnO, as reported by Conway et al. [20]. Furthermore, MP reduced ADG compared to the ZnO group, and MPD_2_ supplementation reduced ADG compared with the control and ZnO group. In contrast, dietary supplementation with MPD2 (*Agaricus bisporus*) improved ADG and FCR in 150-day old finisher pigs [19] and mushroom vitamin D_2_ was 77% as effective in raising serum total 25-OH-D concentration as vitamin D_3._

It is important to note that the study by Duffy et al. [19] was conducted in the same unit as the current study, using a similar genotype pig, and thus we hypothesized that the discrepancy in results is due to the difference in the age and maturity of the pigs used in each study. Zinc oxide supplementation had no effect on the growth performance of the pigs compared to the control. However, this effect of ZnO may have been more pronounced in a commercial farm setting.

The negative effect of MP and MPD_2_ on feed intake and performance in the weaned pigs was considered a negative outcome in this study. The mechanism behind the reduced feed intake was not explored in this study but is worthy of further consideration. It is well known that mushrooms contain a spectrum of compounds that have biological activity and could influence feed intake either through a satiety effect and/or a reduced palatability effect.

These bioactive compounds include polyphenols and phytochemicals, such as tannins, saponins and phytates [45], which can affect the palatability of feed due to their astringent taste, consequently depressing feed intake. Furthermore, the non-digestible polysaccharides present in mushrooms, including chitin [46], have been associated with increased satiety. Agaricus bisporus mushrooms contain chitin concentrations of approximately 7.8% DM [47], and supplementation of this mushroom variety reduced feed intake in broilers [48].

Dietary chitosan, the deacetylated form of chitin, influences feed intake and the expression of associated adipokines and genes in pigs [49,50,51]. Thus, the reduction in feed intake in this study may be attributed to the mushrooms having a satiety effect and/or reduced palatability and could be a valuable mechanism of reducing feed intake in other scenarios.

A lack of enteral nutrition leads to adverse morphological changes, such as villous atrophy and crypt hyperplasia, occurring in the gastrointestinal tract [52]. It is interesting to note that, in this study, while there is evidence to suggest that the MP and MPD_2_ supplemented animals had improved morphological characteristics and increased expression of the peptide transporter *SLC15A1* and fatty acid binding protein *FABP2* in the duodenum, feed intake was reduced compared with the control group.

In a previous study, chicks supplemented with mushrooms had increased VH in the ileum despite an overall reduction in FI and performance [53]. Furthermore, β-glucan supplementation enhanced the villus height in chickens, as well as restored some of the villus damage caused by a Salmonella challenge [54].

The observed improvement in gastrointestinal health consequently enhanced the digestive and absorptive capacity of the gastrointestinal epithelium resulting in an upregulation of nutrient transporters in the duodenum. These results indicate that MP and MPD_2_ supplementation has the potential to support gastrointestinal health through the improvement of small intestinal architecture in the post-weaning period.

Gastrointestinal homeostasis is of utmost importance to the health of the weaned pig and disruption to this gives rise to intestinal inflammation. Inflammation is associated with elevated levels of inflammatory cytokines, such as TNF-α, IL-6, IL-1β and IL-8, throughout the intestine, as observed during the immediate post-weaning period [55]. In the present study, pigs offered the MPD_2_ diet had increased expression of vitamin D receptor *VDR* in the duodenum compared with pigs offered the control and ZnO diets.

Thus, although not measured, the increased expression of *VDR* in the duodenum is likely a result of higher serum total 25-OH-D concentration in the mushroom supplemented pigs. Furthermore, MPD_2_ decreased the expression of *IL6* and increased the expression of *IL10*; indicating its immunomodulatory potential and ability to resolve inflammation. In the duodenum, MPD_2_ decreased the expression of *IL6* compared with pigs offered the control diet. IL-6 is a pro-inflammatory cytokine, expressed during states of inflammation and infection.

It is involved in the production of acute phase proteins and is associated with increased epithelial permeability [2,56]. In the ileum, the expression of *IL10* was increased when pigs were offered MPD_2_ compared with pigs offered the control and ZnO diets. IL-10 is considered a potent anti-inflammatory cytokine due to its ability to reduce Th-1 and Th-2 cytokine production and its potential to promote the resolution of inflammation [57].

The microbial composition in the large intestine of pigs can be positively influenced via dietary interventions to improve animal productivity and health and to inhibit colonization by enteric pathogens. Bacterial members of the genus *Lactobacillus* can enhance host gastrointestinal health through the competitive exclusion of pathogenic bacteria, producing antimicrobial peptides, such as bacteriocins, and aiding regulation of the immune system [58,59].

Zinc oxide supplementation resulted in an increase in the relative abundance of *Lactobacillus* (Firmicutes phylum, *Lactobacillaceae* family) compared with the control, MP and MPD_2_ groups. Interestingly, ZnO (2500 mg/kg) supplementation had no effect on *Lactobacillus* counts at week 1 and 3 of supplementation; however, *Lactobacillus* counts were increased at week 6 [60].

Furthermore, ZnO (2000 mg Zn/kg feed) supplementation enhanced the relative abundance of *Lactobacillus*; however, supplementing ZnO at levels exceeding this inclusion rate had detrimental effects on the relative abundance of *Lactobacillus* [3]. It may be possible that the removal of ZnO from the diet for a period of 10 days enabled the recovery of these bacteria, and thus an increased abundance of *Lactobacillus* was observed in the ZnO group.

It was anticipated that MP and MPD_2_ supplementation would increase beneficial microbial populations based on previous studies with *A*. *bisporus* mushroom and Vitamin D [11,61,62]. However, based on the 16S rRNA gene sequencing, MP and MPD_2_ supplementation did not positively influence the lactobacilli counts compared with the control or that of ZnO supplemented pigs. In previous studies, *Agaricus bisporus* mushroom supplementation increased lactobacilli counts in the ileum and caecum of chickens [11] and turkeys [12].

The discrepancy between studies may be attributed to the reduced feed intake observed in the present study. Bacteria in the large intestine are dependent on dietary substrates for energy and survival and thus, the observed reduced feed intake may have disrupted the continuous delivery of substrate, resulting in decreased energy sources for the host animal and its microbiota. A higher abundance of *Prevotella* has been positively correlated with feed intake [63,64] and body weight [65] as well as acetic, butyric, propionate and total VFA production [66].

In the present study, the supplementation of MP, MPD_2_ and ZnO resulted in a decrease in the relative abundance of *Prevotella* (Bacteroidetes phylum, *Prevotellaceae* family) compared with the control group. The concentrations of acetate, butyrate and propionate were unaffected in our study; however, the concentration of total VFAs in the colon was increased by MPD_2_ supplementation but unaffected by MP and ZnO supplementation.

## 5. Conclusions

Dietary supplementation with vitamin D_2_-enriched mushroom powder improved gastrointestinal morphology, increased total volatile fatty acid concentrations, increased expression of anti-inflammatory cytokine and nutrient transporter genes and reduced the expression of pro-inflammatory cytokine genes. The mushroom powder supplementation had a positive impact on the gastrointestinal morphology and upregulated the expression of genes associated with nutrient transport and gastrointestinal integrity but had no effect on the expression of inflammatory markers, suggesting that vitamin D_2_-enriched mushroom powder and mushroom powder have differing modes of action.

It is noteworthy that vitamin D_2_-enriched mushroom powder was equally as effective as zinc oxide in improving gastrointestinal health parameters; however, its negative effects on feed intake translated to a reduction in animal performance. As mushroom powder and vitamin D_2_-enriched mushroom powder have a negative effect on feed intake and performance, they have limited use as a commercial feed additive in replacing zinc oxide in post-weaned pig diets.

## Figures and Tables

**Table 1:** Ingredients and chemical compositions of the diets.

	Treatments ^1^			
Ingredients (g/kg unless Otherwise Stated)	Basal	Zinc Oxide	Mushroom	Vit D_2_ Mushroom
Ground wheat	355.4	352.3	353.4	353.4
Full-fat soya bean	170	170	170	170
Soya bean meal (48% CP)	105	105	105	105
Whey powder (900 g CP/kg)	50	50	50	50
Mushroom Powder	0	0	2	2
Zinc oxide	0	3.1	0	0
Soya oil	30	30	30	30
Soybean concentrate	65	65	65	65
Flaked wheat	130	130	130	130
Flaked maize	70	70	70	70
L-Lysine-HCl	4	4	4	4
DI-Methionine	2	2	2	2
L-Threonine	1.8	1.8	1.8	1.8
L-Tryptophan	0.3	0.3	0.3	0.3
Sodium bicarbonate	2	2	2	2
Monocalcium phosphate	4	4	4	4
Vitamin and mineral premix ^2^	2.5	2.5	2.5	2.5
Calcium carbonate (limestone)	6	6	6	6
Salt	2	2	2	2
Analysed chemical analysis				
Gross energy (MJ/kg)	16.9	16.9	16.8	16.9
Dry matter	899	899.5	897.5	898.1
Crude protein	208	208.3	208.5	208.5
Lysine (%) ^3^	1.4	1.4	1.4	1.4
Threonine (%) ^3^	0.9	0.9	0.9	0.9
Methionine and cysteine (%) ^3^	0.8	0.8	0.8	0.8
Tryptophan (%) ^3^	0.3	0.3	0.3	0.3
Standardised ileal digestible lysine ^3^	13.0	13.0	13.0	13.0
Crude fat	79.9	80.3	80.1	80
Crude fibre	28	28	28.2	28.3
Neutral detergent fibre	99	98.7	99.5	99.3
Ash	46.2	46.1	46	46.2
Vitamin D_3_ (µg/kg) ^3^	50	50	50	50
Additional vitamin D_2_ (µg/kg)	0	0	0	99
Β-glucan (mg/kg)	0	0	198	205

^1^ Treatments: (1) basal diet; (2) basal diet + ZnO (3100 mg/kg d 1–14, 1550 mg/kg d 15–35 and withdrawn entirely d 36–45); (3) basal diet + mushroom powder (2 g/kg feed) containing 200 mg/kg β-glucan; (4) basal diet + Vitamin D_2_-enriched mushroom powder (2 g/kg feed) containing 200 mg/kg β-glucan with 100 ug/kg additional vitamin D2. ^2^ Provided (per kg diet): 1.8 mg retinol; 0.025 mg cholecalciferol; 67 mg tocopherol; 4 mg menaquinone; 0.1 mg cyanocobalamin; 2 mg riboflavin; 12 mg nicotinic acid; 10 mg pantothenic acid; 250 mg choline chloride; 2 mg thiamine; 0.015 mg pyridoxine; 25 mg copper; 140 mg iron; 47 mg Manganese; 120 mg Zinc; 0.6mg iodine; 0.3 mg sulphur. ^3^ Calculated for the tabulated nutritional composition [25].

**Table 2:** Panel of porcine oligonucleotide primers used for real-time PCR.

Target Gene	Accession No.	Forward Primer (5’-3’) Reverse Primer (5’-3’)	Amplicon Length (bp)
*IL6*	NM_214399.1	F: GACAAAGCCACCACCCCTAA R: CTCGTTCTGTGACTGCAGCTTATC	69
*CXCL8*	NM_213867.1	F: TGCACTTACTCTTGCCAGAACTG R: CAAACTGGCTGTTGCCTTCTT	82
*IL10*	NM_214041.1	F: GCCTTCGGCCCAGTGAA R: AGAGACCCGGTCAGCAACAA	71
*IL17A*	NM_001005729.1	F: CCCTGTCACTGCTGCTTCTG R: TCATGATTCCCGCCTTCAC	57
*IFNG*	NM_213948.1	F: TCTAACCTAAGAAAGCGGAAGAGAA R: TTGCAGGCAGGATGACAATTA	81
*TNF*	NM_214022.1	F: TGGCCCCTTGAGCATCA R: CGGGCTTATCTGAGGTTTGAGA	68
*TGFB1*	NM_214015.1	F: AGGGCTACCATGCCAATTTCT R: CGGGTTGTGCTGGTTGTACA	101
*TLR4*	NM_001293317.1	F: TGCATGGAGCTGAATTTCTACAA R: GATAAATCCAGCACCTGCAGTTC	140
*MUC1*	XM_001926883.1	F: ACACCCATGGGCGCTATGT R: GCCTGCAGAAACCTGCTCAT	68
*MUC2*	XM_021082584.1	F: CAACGGCCTCTCCTTCTCTGT R: GCCACACTGGCCCTTTGT	70
*ZOI*	XM_005659811.1	F: TGAGAGCCAACCATGTCTTGAA R: CTCAGACCCGGCTCTCTGTCT	76
*CLND3*	NM_001160075.1	F: GAGGGCCTGTGGATGAACTG R: GAGTCGTACACTTTGCACTGCAT	65
*CCK*	NM_214237.2	F: GGACCCCAGCCACAGAATAA R: GCGCCGGCCAAAATC	
*FABP2*	NM_001031780.1	F: CAGCCTCGCAGACGGAACTGAA R: GTGTTCTGGGCTGTGCTCCAAGA	102
*SLC2A1/GLUT1*	XM_003482115.1	F: TGCTCATCAACCGCAATGA R: GTTCCGCGCAGCTTCTTC	72
*SLC2A2/GLUT2*	NM_001097417.1	F: CCAGGCCCCATCCCCTGGTT R: GCGGGTCCAGTTGCTGAATGC	96
*SLC2A5/GLUT5*	XM_021095282.1	F: CCCAGGAGCCGGTCAAG R: TCAGCGTCGCCAAAGCA	60
*SLC5A1/* *SGLT1*	NM_001164021	F: GGCTGGACGAAGTATGGTG R: ACAACCACCCAAATCAGAGC	
*SLC15A1/PEPT1*	NM_214347.1	F: GGATAGCCTGTACCCCAAGCT R: CATCCTCCACGTGCTTCTTGA	73
*VDR*	NM_001097414.1	F: CCTTCACCATGGACGACATG R:TGGCCACGTCGCTGACTT	73
*B2M*	NM_213978.1	F: CGGAAAGCCAAATTACCTGAAC R:TCTCCCCGTTTTTCAGCAAAT	83
*GAPDH*	NM_001206359	F: CAGCAATGCCTCCTGTACCA R: ACGATGCCGAAGTTGTCATG	72
*PPIA*	NM_214353.1	F: CGGGTCCTGGCATCTTGT R: TGGCAGTGCAAATGAAAAACT	75
*YWZHAZ*	NM_001315726.1	F: GGACATCGGATACCCAAGGA R:AAGTTGGAAGGCCGGTTAATTT	71

**Table 3:** Effect of dietary treatment on pig growth performance (day 1–45; least square means with their standard errors).

	Dietary Treatments ^1^				SEM	*p* Values
	Control ^2^	ZnO ^2^	Mushroom ^2^	Vit D_2_ Mushroom ^2^		Treatment
Initial body weight (kg)	7.8	7.8	7.7	7.8	0.16	0.824
Final body weight (kg)	31.3 ^b,c^	31.8 ^c^	30.4 ^a,b^	29.5 ^a^	0.49	0.007
ADG (kg/day)	0.56 ^b,c^	0.57 ^c^	0.54 ^a,b^	0.52 ^a^	0.012	<0.001
ADFI (kg/day)	0.91 ^b^	0.96 ^c^	0.87 ^a^	0.88 ^a^	0.009	<0.001
Gain:feed	0.62	0.61	0.62	0.60	0.013	0.723
Faecal score^3^	3.00 ^b^	2.57 ^a^	3.07 ^b^	3.00 ^a^	0.067	<0.001
Diarrhoea incidence % (day 1–14) ^*^	25.0 ^a^	2.8 ^b^	38.9 ^a^	36.1 ^a^	0.136	<0.001

ADG, average daily gain; ADFI, average daily feed intake. ^1^ Treatments: (1) basal diet; (2) basal diet + ZnO (3100 mg/kg d 1–14, 1550 mg/kg d 15–35 and withdrawn entirely d 36–45); (3) basal diet + mushroom powder (2 g/kg feed) containing 200 mg/kg β-glucan; (4) basal diet + Vitamin D_2_-enriched mushroom powder (2 g/kg feed) containing 200 mg/kg β-glucan with 100 ug/kg additional vitamin D_2_. ^a–c^ Mean values within a row with unlike superscript letters were significantly different (*p* < 0.05). ^2^ A total of twelve replicates were used per treatment group (experimental unit = pen). * A faecal score of greater than 3 was categorized as diarrhoea. ^3^ Faecal score range: 1 = hard, firm faeces; 2 = slightly soft faeces; 3 = soft, partially formed faeces; 4 = loose, semi-liquid faeces; and 5 = watery, mucous like faeces.

**Table 4:** Effect of dietary treatment on villus height and crypt depth in the small intestine (least square means with their standard errors).

Intestinal Site		Treatments ^1^				SEM	*p*-Values
		Control ^2^	ZnO ^2^	Mushroom ^2^	Vit D_2_ Mushroom ^2^		
Duodenum	VH µm	342.5 ^a^	391.6 ^b^	395.8 ^b^	381.4 ^b^	14.61	<0.05
	CD µm	163.6	170.4	169.0	177.2	8.38	0.719
	VH:CD	2.1	2.4	2.4	2.2	0.14	0.446
Jejunum	VH µm	288.0 ^a^	319.6 ^b^	302.3 ^a,b^	318.9 ^b^	10.52	0.12
	CD µm	167.3 ^b^	159.5 ^b^	133.6 ^a^	149.7 ^a,b^	9.02	0.064
	VH:CD	1.8 ^a^	2.1 ^a,b^	2.3 ^b^	2.2 ^b^	0.14	0.068
Ileum	VH µm	289.7 ^a^	322.1 ^b^	306.8 ^a,b^	322.3 ^b^	10.14	<0.05
	CD µm	167.3 ^b^	159.5 ^b^	133.6 ^a^	149.7 ^a,b^	9.01	<0.05
	VH:CD	1.8 ^a^	2.1 ^a,b^	2.4 ^b^	2.2 ^b^	0.14	0.059

VH, villus height; CD, crypt depth; VH:CD, villus height to crypt depth ratio. ^1^ Treatments: (1) basal diet; (2) basal diet + ZnO (3100 mg/kg d 1–14, 1550 mg/kg d 15–35 and withdrawn entirely d 36–45); (3) basal diet + mushroom powder (2 g/kg feed) containing 200 mg/kg β-glucan; and (4) basal diet + Vitamin D_2_-enriched mushroom powder (2 g/kg feed) containing 200 mg/kg β-glucan with 100 ug/kg additional vitamin D_2_. ^a,b^ Mean values within a row with unlike superscript letters were significantly different (*p* < 0.05). ^2^ A total of 12 replicates were used per treatment group (experimental unit = pig).

**Table 5:** The effect of dietary treatment on measures of alpha diversity (least-square means with their standard errors).

	Treatments ^1^				SEM	*p*-Values
	Control ^2^	ZnO ^2^	Mushroom ^2^	Vit D_2_ Mushroom ^2^		
Observed	23.00	24.00	25.00	26.00	2.021	0.237
Chao1	55.42	53.5	50.58	52.54	0.888	0.723
ACE	22.37	20.87	24.27	25.72	1.593	0.517
Shannon	6.84	5.89	6.93	6.51	0.208	0.272
Simpson	3.76	3.73	3.61	3.68	0.027	0.479
InvSimpson	0.97	0.97	0.96	0.97	0.001	0.388
Fisher	32.53	31.23	27.73	30.54	1.037	0.301

^1^ Treatments: (1) basal diet; (2) basal diet + ZnO (3100 mg/kg d 1–14, 1550 mg/kg d 15–35 and withdrawn entirely d 36–45); (3) basal diet + mushroom powder (2 g/kg feed) containing 200 mg/kg β-glucan; and (4) basal diet + Vitamin D_2_-enriched mushroom powder (2 g/kg feed) containing 200 mg/kg β-glucan with 100 ug/kg additional vitamin D_2_. ^2^ A total of 12 replicates were used per treatment group (experimental unit = pig).

**Table 6:** The effect of dietary treatment on the % bacterial abundance at phylum level (Least-square means with their standard errors).

	Treatment ^1^				SEM	*p*-Values
Phylum	Control ^2^	ZnO ^2^	Mushroom ^2^	Vit D_2_ Mushroom ^2^		
Bacteroidetes	33.4	29.07	30.08	29.74	1.596	0.239
Firmicutes	64.72	68.76	67.8	67.84	2.368	0.649
Tenericutes	0.11	0.04	0.17	0.06	0.085	0.763
Actinobacteria	0.51	0.72	0.63	0.5	0.221	0.88
Proteobacteria	0.54	1.03	0.32	1.01	0.24	0.15

^1^ Treatments: (1) basal diet; (2) basal diet + ZnO (3100 mg/kg d 1–14, 1550 mg/kg d 15–35 and withdrawn entirely d 36–45); (3) basal diet + mushroom powder (2 g/kg feed) containing 200 mg/kg β-glucan; and (4) basal diet + Vitamin D_2_-enriched mushroom powder (2 g/kg feed) containing 200 mg/kg β-glucan with 100 ug/kg additional vitamin D_2_. ^2^ A total of twelve replicates were used per treatment group (experimental unit = pig).

**Table 7:** The effect of dietary treatment on the % bacterial abundance at family level (Least-square means with their standard errors).

	Treatments ^1^			SEM		*p*-Values
Family	Control ^2^	ZnO ^2^	Mushroom ^2^	Vit D_2_ Mushroom ^2^		
Prevotellaceae	33.10 ^b^	27.84 ^a^	29.65 ^a,b^	29.30 ^a,b^	1.58	0.130
Muribaculaceae	0.11	0.46	0.14	0.14	0.126	0.273
Propionibacteriaceae	0.03	0.12	0.01	0.03	0.057	0.728
Burkholderiaceae	0.12	0.28	0.12	0.14	0.115	0.734
Tannerellaceae	0.17	0.3	0.09	0.03	0.103	0.466
Lachnospiaceae	15.87	14.65	13.91	16.47	1.126	0.376
Eubacteriaceae	0.49	0.44	0.48	0.43	0.196	0.995
Ruminococcaceae	23.67	23.61	22.36	23.06	1.390	0.902
Peptostreptococcaceae	0.28	0.31	0.18	0.36	0.152	0.863
VAR15	3.92	5.16	5.39	4.72	0.631	0.388
Hungateiclostridiaceae	0.16	0.27	0.08	0.15	0.114	0.735
Clostridiaceae	3.17	3.66	4.14	3.90	0.556	0.643
Oscillospiraceae	0.05	0.15	0.10	0.04	0.080	0.791
Mycoplasmataceae	0.06	0.02	0.17	0.06	0.075	0.67
Coriobacteriaceae	0.38	0.39	0.53	0.40	0.188	0.93
Enterobacteriaceae	0.03	0.41	0.04	0.33	0.114	0.238
Succinivibrionaceae	0.38	0.19	0.16	0.39	0.149	0.615
Veillonellaceae	0.7	0.39	0.65	0.61	0.220	0.772
Selenomonadaceae	1.1	1.02	1.22	1.27	0.31	0.934
Streptococcaceae	4.41	4.6	5.68	5.94	0.654	0.271
Christensenellaceae	0.02	0.02	0.08	0.03	0.053	0.852
Erysipelotrichaceae	0.04	0.11	0.07	0.23	0.093	0.593
Lactobacillaceae	10.58 ^a^	14.16 ^b^	9.92 ^a^	10.32 ^a^	1.000	0.022

^1^ Treatments: (1) basal diet; (2) basal diet + ZnO (3100 mg/kg d 1–14, 1550 mg/kg d 15–35 and withdrawn entirely d 36–45); (3) basal diet + mushroom powder (2 g/kg feed) containing 200 mg/kg β-glucan; and (4) basal diet + Vitamin D_2_-enriched mushroom powder (2 g/kg feed) containing 200 mg/kg β-glucan with 100 ug/kg additional vitamin D_2_. ^a,b^ Mean values within a row with unlike superscript letters were significantly different (*p* < 0.05). ^2^ A total of twelve replicates were used per treatment group (experimental unit = pig).

**Table 8:** The effect of dietary treatment on the % bacterial abundance at genus level (Least-square means with their standard errors).

	Treatments ^1^				SEM	*p*-Values
Genus	Control ^2^	ZnO ^2^	Mushroom ^2^	Vit D_2_ Mushroom ^2^		
Prevotella	29.06 ^b^	23.38 ^a^	24.90 ^a^	23.94 ^a^	1.451	0.035
Muribaculum	0.11	0.39	0.13	0.12	0.119	0.363
Propionibacterium	0.03	0.12	0.01	0.03	0.057	0.728
Ralstonia	0.12	0.28	0.12	0.14	0.115	0.734
Parabacteroides	0.17	0.30	0.09	0.03	0.103	0.466
Alloprevotella	0.94	1.16	1.25	1.15	0.306	0.905
Prevotellamassilia	3.05	3.30	3.51	4.2	0.540	0.482
Oribacterium	0.32	0.30	0.24	0.22	0.149	0.956
Anaerobutyricum	0.79	0.78	0.61	0.79	0.248	0.942
Dorea	1.75	1.70	1.86	1.85	0.386	0.988
Coprococcus	2.7	2.65	2.92	3.89	0.502	0.282
Lachnoclostridium	0.35	0.09	0.06	0.24	0.117	0.395
Anaerostipes	0.21	0.12	0.08	0.09	0.100	0.782
Blautia	1.39	1.74	1.53	1.52	0.358	0.919
Lachnobacterium	0.07	0.04	0.02	0.06	0.060	0.95
Roseburia	3.29	2.79	3.01	3.42	0.510	0.817
Pseudobutyrivibrio	2.89	2.20	1.91	2.99	0.454	0.284
Eubacterium	0.49	0.44	0.48	0.43	0.196	0.995
Kineothrix	0.14	0.31	0.15	0.09	0.117	0.642
Murimonas	0.38	0.38	0.38	0.38	0.172	0.744
Gemmiger	5.58	5.29	5.17	6.92	0.690	0.271
Faecalibacterium	15.80	15.40	15.33	13.29	1.116	0.392
Peptoclostridium	0.27	0.31	0.15	0.32	0.146	0.856
Clostridium	1.24	1.35	2.04	1.87	0.366	0.356
Anaerobacterium	0.15	0.26	0.08	0.12	0.110	0.703
Ruminococcus	1.31	1.52	1.06	1.42	0.332	0.783
Neglecta	0.03	0.04	0.02	0.07	0.056	0.946
Agathobaculum	0.39	0.50	0.28	0.55	0.188	0.764
Butyricicoccus	1.93	2.31	2.11	2.03	0.417	0.932
Oscillibacter	0.05	0.15	0.10	0.04	0.112	0.791
Sporobacter	0.20	0.43	0.19	0.43	0.158	0.589
Intestinimonas	0.09	0.13	0.26	0.18	0.115	0.784
Mycoplasma	0.06	0.02	0.17	0.06	0.075	0.670
Succinivibrio	0.38	0.19	0.16	0.39	0.149	0.615
Megasphaera	0.15	0.17	0.19	0.13	0.114	0.984
Dialister	0.55	0.22	0.46	0.48	0.187	0.657
Mitsuokella	0.04	0.03	0.03	0.01	0.047	0.983
Anaerovibrio	1.06	0.99	1.19	1.26	0.306	0.920
Lactococcus	0.01	0.01	0.03	0.02	0.038	0.968
Streptococcus	4.40	4.59	5.64	5.92	0.653	0.282
Beduinibacterium	0.02	0.02	0.08	0.03	0.053	0.852
Turicibacter	0.04	0.11	0.07	0.23	0.093	0.593
Lactobacillus	10.58 ^a^	14.16 ^b^	9.92 ^a^	10.32 ^a^	1.000	0.022

^1^ Treatments: (1) basal diet; (2) basal diet + ZnO (3100 mg/kg d 1–14, 1550 mg/kg d 15–35 and withdrawn entirely d 36–45); (3) basal diet + mushroom powder (2 g/kg feed) containing 200 mg/kg β-glucan; and (4) basal diet + Vitamin D_2_-enriched mushroom powder (2 g/kg feed) containing 200 mg/kg β-glucan with 100 ug/kg additional vitamin D_2_. ^a–b^ Mean values within a row with unlike superscript letters were significantly different (*p* < 0.05). ^2^ A total of twelve replicates were used per treatment group (experimental unit = pig).

**Table 9:** The effect of dietary treatment on the total concentrations and molar proportions of VFA in the colon (least-square means with their standard errors).

	Treatments ^1^				SEM	*p*-Value
	Control ^2^	ZnO ^2^	Mushroom ^2^	Vit D_2_ Mushroom ^2^		
Total VFA (mmol/g digesta)	185.25 ^a^	172.10 ^a^	194.00 ^a^	231.73 ^b^	12.684	0.014
Molar proportions (%)						
Acetate	68.40	71.39	69.43	71.28	1.244	0.262
Propionate	21.54	19.80	20.83	20.11	0.763	0.390
Butyrate	7.49 ^a,b^	7.75 ^b^	7.71 ^a,b^	6.47 ^a^	0.405	0.145
Isovalerate	0.58 ^a,b^	0.70 ^a^	0.63 ^a^	0.41 ^b^	0.076	0.069
Isobutyrate	0.36 ^b^	0.37 ^b^	0.32 ^a,b^	0.24 ^a^	0.039	0.116
Valerate	1.62	1.63	1.68	1.22	0.183	0.270

^1^ Treatments: (1) basal diet; (2) basal diet + ZnO (3100 mg/kg d 1–14, 1550 mg/kg d 15–35 and withdrawn entirely d 36–45); (3) basal diet + mushroom powder (2 g/kg feed) containing 200 mg/kg β-glucan; and (4) basal diet + Vitamin D_2_-enriched mushroom powder (2 g/kg feed) containing 200 mg/kg β-glucan with 100 ug/kg additional vitamin D_2_. ^a,b^ Mean values within a row with unlike superscript letters were significantly different (*p* < 0.05). ^2^A total of 12 replicates were used per treatment group (experimental unit = pig).

**Table 10:** The effects of dietary treatment on the expression of nutrient transporters, immune markers and tight junction genes in the duodenum and ileum; the expression of appetite regulators in the duodenum and ileum (least square means with their standard errors).

		Treatments ^1^				SEM	*p*-Value
	Gene	Control ^2^	ZnO ^2^	Mushroom ^2^	Vit D_2_ Mushroom ^2^		
Duodenum							
Nutrient transporters	*SLC15A1/PEPT1*	0.81 ^a^	1.22 ^b^	1.20 ^b^	1.10 ^b^	0.090	0.012
	*FABP2*	0.79 ^a^	1.31 ^b^	1.13 ^b^	1.19 ^b^	0.119	0.026
	*VDR*	0.94 ^a^	0.94 ^a^	1.11 ^a,b^	1.22 ^b^	0.095	0.122
	*SLC2A1/GLUT1*	1.16	0.98	1.08	1.10	0.117	0.766
Tight junctions and immune markers	*TNF*	1.16 ^a^	0.78 ^b^	1.03 ^a, b^	0.99 ^a,b^	0.119	0.174
	*CXCL8*	0.90 ^a^	1.21 ^b^	1.05 ^a,b^	0.97 ^a^	0.099	0.163
	*IL6*	1.22 ^a^	0.72 ^b^	1.05 ^a,b^	0.78 ^b^	0.153	0.098
	*IL10*	1.15	1.01	0.92	1.04	0.120	0.629
	*IFNG*	1.13	0.99	1.06	0.98	0.112	0.807
	*ZO1*	1.02	1.02	1.09	0.95	0.062	0.465
	*MUC2*	1.19	1.00	1.22	0.97	0.144	0.497
Ileum							
Nutrient transporters	*FABP2*	1.40	1.00	1.35	1.16	0.235	0.611
	*SLC15A1/PEPT1*	1.36	0.81	1.23	1.19	0.205	0.276
	*VDR*	1.39	1.09	1.12	1.10	0.241	0.804
	*SLC5A1/SGLT1*	1.33	0.87	1.28	1.43	0.192	0.189
	*SLC2A1/GLUT1*	1.36	0.90	0.92	1.03	0.136	0.087
	*SLC2A2/GLUT2*	1.80 ^a^	0.71 ^b^	1.31 ^a,b^	1.37 ^a,b^	0.273	0.06
	*SLC2A5/GLUT5*	1.61	1.03	1.14	1.17	0.276	0.517
Tight junctions and immune markers	*TNF*	2.98	1.21	1.09	1.13	0.126	0.652
	*CXCL8/IL8*	0.98 ^a,b^	1.12 ^a,b^	0.86 ^a^	1.19 ^b^	0.114	0.191
	*IL6*	0.97	1.24	1.16	1.32	0.170	0.532
	*IL10*	0.89 ^a^	0.96 ^a^	1.17 ^a,b^	1.34 ^b^	0.131	0.078
	*TGFB*	0.98	1.12	1.06	1.13	0.109	0.772
	*IL17*	0.90	1.43	1.05	1.23	0.197	0.274
	*TLR4*	1.02	0.95	1.14	1.21	0.110	0.337
	*CLDN3*	1.82	1.11	1.19	1.35	0.292	0.341
	*CLDN1*	1.03 ^a^	1.00 ^a^	1.40 ^b^	0.86 ^a^	0.129	0.032
	*MUC1*	1.4	1.03	0.91	1.26	0.195	0.298
	*MUC2*	1.36	1.04	1.3	1.56	0.261	0.578
Appetite regulators	*CCK*	0.95	1.07	0.79	1.02	0.127	0.389

*SLC15A1/PEPT1*, peptide transporter 1; *FABP2*, fatty acid binding protein 2; *VDR*, vitamin d receptor; *SLC2A1/GLUT1*, glucose transporter 1; *SLC5A1/SGLT1*, sodium glucose linked transporter 1; *SLC2A2/GLUT2*, glucose transporter 2; *SLC2A5/GLUT5*, glucose transporter 5; *CCK*, cholecystokinin; *TNF*, tumour necrosis factor alpha; *CXCL8*, interleukin 8; *IL6*, interleukin 6; *IL10*, interleukin 10; *IFNG*, interferon gamma; *ZO1*, zonulin; *MUC2*, mucin 2; TGF-β, transforming growth factor beta; *IL17*, interleukin 17; *TLR4*, toll like receptor 4; *CLDN3*, claudin 3; *CLDN1*, claudin 1; *MUC1*, mucin 1. ^1^ Treatments: (1) basal diet; (2) basal diet + ZnO (3100 mg/kg d 1–14, 1550 mg/kg d 15–35 and withdrawn entirely d 36–45); (3) basal diet + mushroom powder (2 g/kg feed) containing 200 mg/kg β-glucan; and (4) basal diet + Vitamin D_2_-enriched mushroom powder (2 g/kg feed) containing 200 mg/kg β-glucan with 100 ug/kg additional vitamin D_2_. ^a,b^ Mean values within a row with unlike superscript letters were significantly different (*p* < 0.05). ^2^ A total of 12 replicates were used per treatment (experimental unit = pig).

## Data Availability

The data presented in this study are available on request from the corresponding author.
